# Spectrum of Viral Infections Among Primary Immunodeficient Children: Report From a National Registry

**DOI:** 10.3389/fimmu.2019.01231

**Published:** 2019-05-29

**Authors:** Waleed Al-Herz, Sahar Essa

**Affiliations:** ^1^Department of Pediatrics, Faculty of Medicine, Kuwait University, Kuwait City, Kuwait; ^2^Allergy and Clinical Immunology Unit, Pediatric Department, Al-Sabah Hospital, Kuwait City, Kuwait; ^3^Department of Microbiology, Faculty of Medicine, Kuwait University, Kuwait City, Kuwait

**Keywords:** primary immunodeficiencies, virus, CMV, EBV, adenovirus, pneumonia, viremia, mortality

## Abstract

**Objective:** To present the frequency and spectrum of viral infections in primary immunodeficient children.

**Methods:** The data was obtained from the Kuwait National Primary Immunodeficiency Disorders (PIDs) Registry during the period of 2004-2018.

**Results:** A total of 274 PID children were registered in KNPIDR during the study period with predominance of immunodeficiencies affecting cellular and humoral immunity, followed by combined immunodeficiencies with associated syndromic features and diseases of immune dysregulation. Overall infectious complications affected 82.4% of the patients, and viral infections affected 31.7% of the registered patients. Forty-five patients (16.4%) developed viral infections caused by at least 2 organisms, among those 20 patients were affected by three or more viral infections. There was a statistically significant association between viral infections and PID category. However, there was no statistically significant association between viral infections and gender or the patients' onset age. There was a total of 170 viral infections during the study period and the causes of these infections were predominated by CMV (22.2%), adenovirus (11.7%), EBV (11.1%), and enteroviruses (7.4%). CMV and parainfluenza infections were more common in the group of immunodeficiencies affecting cellular and humoral immunity while EBV and human papilloma virus (HPV) were more common in the immune dysregulation group and combined immunodeficiencies with associated syndromic features, respectively. The most common presentation was viremia (28.8%) followed by pneumonia (28.2%) and skin infections (17.6%). The most common causes of viremia were CMV followed by adenovirus and EBV, while the most common organisms causing pneumonia were CMV followed by rhinovirus and parainfluenza. There were 80 deaths among the registered patients, 10% were caused by viral infections.

**Conclusions:** Viral infections are common in PIDs and result into a wide-range of clinical manifestations causing significant morbidity and mortality.

## Introduction

Primary immunodeficiency disorders (PIDs) are monogenic defects affecting the innate and/or adaptive immune systems (1). Patients are at increased risk of a wide range of manifestations including autoimmunity, immune dysregulation, and malignancies, but infectious complications are the commonest (2–4). Historically, PID patients used to die before recognition because of infections due to the lack of effective measures to either prevent or treat them. Advances in public health and the discovery of antimicrobial agents made the diagnosis of PIDs possible (5).

Although therapeutic interventions like intravenous immunoglobulins and hematopoietic stem cell transplants have helped to decrease morbidity and mortality, physicians caring for PID patients frequently struggle with treating infections which are usually recurrent or chronic, severe and are frequently caused by opportunistic organisms. Among these microbes are viruses that in PID patients can be challenging with an increased risk of mortality and may predispose to malignancies (6–8). PIDs may also lead to reduced clearance and prolonged shedding of certain viruses like rhinovirus and poliovirus (9, 10). While most PIDs predispose to a wide spectrum of viral infections, certain diseases enhance vulnerability to specific viral infections (11).

Whereas, the risk of viral infections in PIDs is well-established and was recently reviewed (12, 13), we are not aware of any report that characterizes such infections in a large cohort of patients with different types of PIDs. In this report, we present the frequency and spectrum of viral infections in primary immunodeficient children from Kuwait between January 2004 and December 2018.

## Materials and Methods

### Patients Data

The data was obtained from the Kuwait National Primary Immunodeficiency Disorders Registry (KNPIDR) which was approved by The Research and Ethics Committee of the Ministry of Health in Kuwait and the Kuwait University Health Sciences Center Ethical Committee in accordance with *the Declaration of Helsinki*. The patients were followed prospectively and classified according to the International Union of Immunological Societies, Primary Immunodeficiency Diseases Committee report on Inborn Errors of Immunity (2017) (1). Secondary immunodeficiencies (drug induced, HIV induced, and immunodeficiency associated with metabolic disorders…etc.), were ruled out by obtaining a detailed history and by performing appropriate testing when these disorders were suspected.

### Diagnosis of Viral Infections

The clinical diagnosis was based on the standard of care depending on the patient's signs and symptoms supported by laboratory and/or radiologic findings. For example, patients who were diagnosed with pneumonia presented with respiratory signs and symptoms associated with radiologic findings. The diagnosis of herpes simplex virus (HSV) keratitis and stomatitis, warts caused by human papilloma virus (HPV), varicella-zoster virus (VZV) and molluscum contagiosum infections was based on clinical evaluation. In-house Polymerase Chain Reaction (PCR) was used initially to test for Cytomegalovirus (CMV), Epstein–Barr virus (EBV), *herpesvirus 6 (*HHV-6), adenovirus, respiratory and gastrointestinal viruses. This was replaced later by commercial kits as indicated below.

### Sample Collection and Transportation

For patients with gastrointestinal manifestations stool or colonic samples were collected. Deep nasopharyngeal aspirate or bronchoalveolar lavage samples were collected from patients with respiratory manifestations using sterile nylon flocked swab or by bronchoscopy and placed in viral transport medium. Serum and cerebrospinal fluid samples were collected for viral infections as required. All samples were labeled and transported the earliest to Virology Laboratory, Faculty of Medicine, Kuwait University for day-to-day routine screening for viral infections and storage.

### Nucleic Acid Extraction

Viral nucleic acid from samples was extracted using an automated MagNa Pure LC 2.0 system (Roche Diagnostic Systems, Branchburg, NJ).

### Multiplex Real-Time RT-PCR

The multiplex real-time one-step reverse transcription PCR (RT-PCR) Fast-Track Diagnostics (FTD) Respiratory Pathogen 21 Assay (Fast-Track Diagnostics Ltd., Sliema, Malta), was performed on LightCycler 480 RT-PCR thermocycler (Roche, Meylan, France) to detect viruses. These include Influenza A (Flu A), Influenza A subtype H1N1 (H1N1), human Rhinovirus (HRV), Influenza B (Flu B), human Coronaviruses NL63 (HCoV-NL63), 229E (HCoV-229E), OC43 (HCoV-OC43), and HKU1 (HCoV-HKU1), human Parainfluenza viruses 1,2, 3, and 4 (HPIV-1, 2, 3 and 4), human Bocavirus (HBoV), human Metapneumovirus (HMPV A/B), Respiratory Syncytial virus (RSVA/B), respiratory human Adenovirus (HAdV), Enterovirus (EV), and human Parechovirus (HPeV).

For viral gastroenteritis FTD®Viral GE kit (Fast-Track Diagnostics Ltd, Sliema, Malta) was used for the detection of Rotavirus, Adenovirus, Astrovirus, Norovirus, and Sapovirus. Stool samples were homogenized. Prior to extraction, a 200 mg aliquot was suspended in 1 ml of nuclease-free water or 1 ml of stool transport and recovery buffer (Roche Diagnostics, Meylan, France). Then the suspension was immediately clarified by centrifugation at 11,000 g for 5 min. According to the manufacturer's instructions RT-PCRs was performed on 10 μl of nucleic acid by using LightCycler 480 RT-PCR thermocycler (Roche, Meylan, France).

CMV, EBV, enterovirus, HAdV, and HHV-6 detection were performed on serum, stool/colonic, respiratory or CSF samples by LightMix®Kits (TIB Molbiol, Berlin, Germany). Amplifications were performed according to the manufacturer's instructions on LightCycler 480 RT-PCR thermocycler (Roche, Meylan, France). The presence of poliovirus RNA in clinical samples was confirmed by one-step reverse transcription-PCR, followed by a direct sequencing of PCR products, as described previously (14).

### Statistical Analysis

Data were processed using IBM SPSS, version 25 (IBM Corporation, Armonk, NY, USA 2017). Pearson's Chi-square test was used to assess the association between two categorical variables. The non-parametric Mann-Whitney *U*-test was applied to assess whether the patients' ages at onset of a symptom of PID have a significant effect on the risk of viral infection. The effect of age at onset was assessed both as quantitative and qualitative variables after dividing them into groups (0–5, 6–11, 12–24, 25–48, > 48 months). The *p* ≤ 0.05 was used as the cut-off level for statistical significance.

## Results

A total of 274 PID children (142 males and 132 females) were registered in KNPIDR during the study period. The distribution of these patients according to PID categories is: immunodeficiencies affecting cellular and humoral immunity, 97 patients (35.4%); combined immunodeficiencies with associated syndromic features, 67 patients (24.5%); predominantly antibody deficiencies, 34 patients (12.4%); diseases of immune dysregulation, 47 patients (17.2%); congenital defects of phagocyte number or function, 17 patients (6.2%); autoinflammatory disorders, 1 patient (0.3%); and complement deficiencies, 11 patients (4%). No patients with defects in innate immunity were registered. Seventy-one patients were treated with hematopoietic stem cell transplant (HSCT) and 141 received intravenous immunoglobulins. It is important to mention that of the reported viral infections occurred prior to HSCT in patients who received such treatment.

Overall infectious complications affected 226 patients (82.4%), and viral infections affected 87 patients (31.7% of the registered patients). Forty-five patients (16.4%) developed viral infections caused by at least 2 organisms, mostly in the category of immunodeficiencies affecting cellular and humoral immunity (31 patients). Among those, 20 patients were affected by three or more viral infections. There was a statistically significant association between viral infections and PID category after excluding patients who belong to congenital defects of phagocyte number or function, autoinflammatory disorders and complement deficiencies due to low numbers (*p* < 0.001) (Table 1). However, there was no statistically significant association between viral infections and gender (*p* = 0.488), or the patients' onset age when assessed both as quantitative and qualitative variables (*p*-values 0.23 and 0.655, respectively).

**Table 1:** The frequency of viral infections according to PID category among 274 children registered in KNPIDR.

**PID categories (number of patients)**	**Number of patients with viral infections**
Immunodeficiencies affecting cellular and humoral immunity (97)	54 (55.6%)
Combined immunodeficiencies with associated or syndromic features (67)	16 (23.8%)
Predominantly antibody deficiencies (34)	6 (17.6%)
Diseases of immune dysregulation (47)	10 (21.2%)
Congenital defects of phagocyte number or function (17)	1 (5.8%)
Complement deficiencies (11)	1 (9.1)
Total	87 (31.7%)

**Figure 1:**
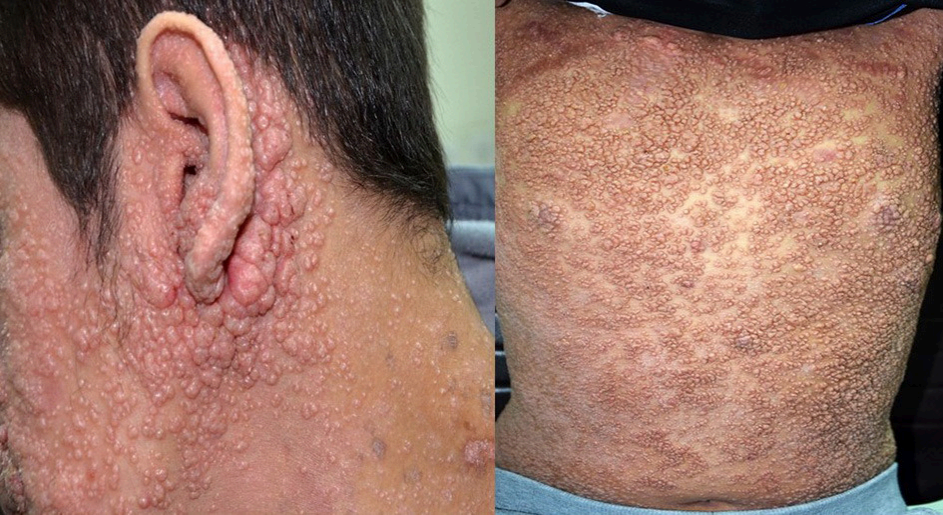
Severe and disseminated molluscum contagiosum in a patient with dedicator of cytokinesis 8 deficiency (DOCK8) deficiency. The patient signed an informed written consent for publication of the images.

**Figure 2:**
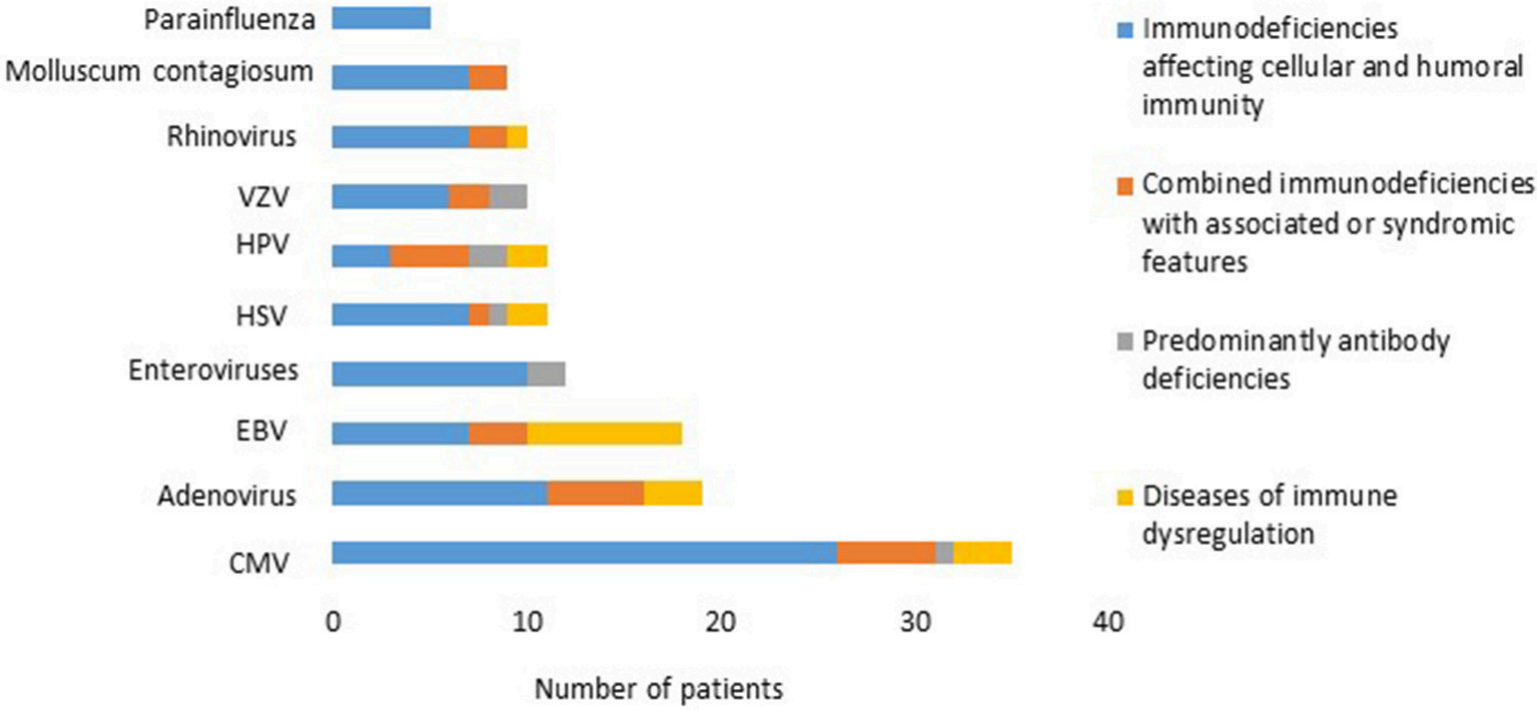
The number of patients affected by different viruses according to PID categories.

There was a total of 170 viral infections during the study period, 33% were detected at the time of PID diagnosis while 67% were documented after establishing the diagnosis. The causes of these infections were: CMV (22.2%); adenovirus (11.7%); EBV (11.1%); enteroviruses (7.4%), HSV and HPV (6.8% each); VZV and rhinovirus (6.2% each); Molluscum contagiosum (5.5%) (Figure 1); norovirus and parainfluenza virus (3% each); H1N1 virus (1.85%); rotavirus, RSV, sapovirus, HHV-6 and corona virus (1.2% each). A patient with severe combined immunodeficiency presented with myocarditis caused by poliovirus type 2. Two patients (1 with severe combined immunodeficiency and 1 with MHC II deficiency) had prolonged excretion of poliovirus type 1 in the stool. The details of the viral infections are presented in Table 2. Figure 2 shows the number of patients affected by different viruses according to PID categories. The most prominent findings are that CMV and parainfluenza infections are more common in the group of immunodeficiencies affecting cellular and humoral immunity while EBV and HPV are more common in the immune dysregulation group and combined immunodeficiencies with associated syndromic features, respectively. The most common presentation was viremia (28.8%) followed by pneumonia (28.2%) and skin infections (17.6%) (Table 3). The most common causes of viremia were CMV followed by adenovirus and EBV, while the most common organisms causing pneumonia were CMV followed by rhinovirus and parainfluenza (Table 3).

**Table 2:** Details of viral infections among the registered patients according to PID diagnosis.

**Diagnosis (number of patients)**	**Viruses isolated (number of patients)**	**Manifestations**
**IMMUNODEFICIENCIES AFFECTING CELLULAR AND HUMORAL**		
**IMMUNITY (*****n*** **=** **54)**		
RAG1 deficiency (5)	CMV (2)	Viremia (1), Retinitis (1), Pneumonia (1)
	EBV (1)	Viremia
	HHV-6 (1)	Viremia
	Rhinovirus (1)	Pneumonia
	Poliovirus-2 (1)	Myocarditis
	Adenovirus (1)	Viremia and liver failure
RAG2 deficiency (4)	EBV (3)	Viremia (3), Meningitis (1)
	CMV (2)	Viremia (2), Pneumonia (1)
	Molluscum contagiosum (2)	Molluscum contagiosum (2)
	Adenovirus (1)	Viremia
	Coronavirus (1)	Pneumonia
DCLRE1C deficiency (4)	Enterovirus (2)	Enteritis (2), Viremia (1)
	CMV (1)	Meningitis
	Rotavirus (2)	Enteritis
	Norovirus (1)	Enteritis
	RSV (1)	Pneumonia
	Rhinovirus (1)	Pneumonia
	Parainfluenza (1)	Pneumonia
JAK3 deficiency (2)	CMV (2)	Viremia (2), Retinitis (1), Pneumonia (1)
AK2 deficiency (3)	CMV (2)	Viremia (1), Colitis (1), Retinitis (1)
	Rhinovirus (1)	Pneumonia
	Adenovirus (1)	Pneumonia
CD3D deficiency (1)	VZV (1)	Chickenpox and pneumonia
	Rhinovirus (1)	Pneumonia
ADA deficiency (1)	Norovirus (1)	Enteritis
DOCK8 deficiency (9)	Molluscum contagiosum (3)	Molluscum contagiosum
	HSV (5)	Stomatitis (5), Keratitis (1)
	VZV (5)	Chickenpox (4), Zoster (1)
	HPV (1)	Warts
	Adenovirus (1)	Viremia
	EBV (1)	Pneumonia
	CMV (4)	Viremia (2), Pneumonia (2)
	Parainfluenza (1)	Pneumonia
DOCK2 deficiency (1)	CMV (1)	Viremia
	Enterovirus (1)	Viremia
MHC II deficiency (8)	RSV (1)	Pneumonia
	CMV (2)	Viremia (1), Pneumonia (1)
	Adenovirus (3)	Viremia (3), Pneumonia (1)
	Poliovirus-1 (1)	Shedding in the stool
	Enterovirus (4)	Viremia (2), Enteritis (2)
	Norovirus (2)	Enteritis (2)
	Molluscum contagiosum (1)	Molluscum contagiosum
	Rhinovirus (1)	Pneumonia
	HHV-6 (1) Parainfluenza (2)	Viremia and meningitis Pneumonia (2)
ZAP70 deficiency (1)	EBV (1)	Viremia
	Rhinovirus (1)	Pneumonia
	CMV (1)	Pneumonia
IKBKB deficiency (1)	Adenovirus (1)	Viremia
	Parainfluenza (1)	Pneumonia
ICOS deficiency (1)	CMV (1)	Viremia
Others (genetically not defined) (13)	HSV (2)	Keratitis (2)
	HPV (2)	Warts (2)
	CMV (8)	Viremia (4), Pneumonia (6)
	Adenovirus (3)	Viremia (2), Pneumonia (1)
	Poliovirus-1 (1)	Shedding in the stool
	Enterovirus (3)	Hemophagocytosis (1), Viremia (1), Pneumonia (1)
	Norovirus (1)	Enteritis
	Molluscum contagiosum (1)	Molluscum contagiosum
	Rhinovirus (1)	Pneumonia
	H1N1 (1)	Pneumonia
	EBV (1)	Viremia
	Coronavirus (1)	Pneumonia
**COMBINED IMMUNODEFICIENCIES WITH ASSOCIATED OR SYNDROMIC**		
**FEATURES (*****n*** **=** **16)**		
Wischott-Aldrich Syndrome (3)	CMV (2)	Viremia (1), Hemophagocytosis (1)
	Molluscum Contagiosum (1)	Molluscum contagiosum
	VZV (1)	Chickenpox
	HSV (1)	Stomatitis
ATM deficiency (3)	HPV (2)	Warts
	Molluscum Contagiosum (1)	Molluscum contagiosum
22q11.2 deletion syndrome (2)	CMV (2)	Pneumonia (1), Viremia (1), Retinitis (1)
STAT3 deficiency (2)	H1N1 (1)	Pneumonia
	VZV (1)	Chickenpox
STAT5B deficiency (2)	Rhinovirus (2)	Pneumonia
	Adenovirus (2)	Viremia
	EBV (2)	Viremia
RMRP deficiency (1)	CMV (1)	Pneumonia
	HPV (1)	Warts
	Adenovirus (1)	Viremia
	EBV (1)	Viremia and lymphoproliferation
HOIP (RNF31) deficiency (1)	HPV (1)	Warts
Immunodeficiency with centromeric instability and facial anomalies (2)	Sapovirus (2)	Enteritis (2)
	Adenovirus (2)	Enteritis (1), Viremia (1)
**PREDOMINANTLY ANTIBODY DEFICIENCIES (*****n*** **=** **6)**		
BTK deficiency (1)	Enterovirus (1)	Meningo-encephalitis
μ Heavy chain deficiency (1)	Enterovirus (1)	Viremia
AICDA deficiency (1)	HSV (1)	Stomatitis
NFKB2 deficiency (1)	HPV (1)	Warts
	CMV (1)	Pneumonia
	VZV (1)	Chickenpox
Common Variable immunodeficiency (1)	HPV (1)	Warts
Transient hypogammaglobulinemia of infancy (1)	VZV (1)	Chickenpox
**DISEASES OF IMMUNE DYSREGULATION (*****n*** **=** **10)**		
LYST deficiency (1)	EBV (1)	Hemophagocytosis
STX11 deficiency (FHL4) (1)	EBV (1)	Hemophagocytosis
STXBP2 deficiency (FHL5) (2)	CMV (2)	Colitis (1), Viremia (2)
	EBV (2)	Viremia
LRBA deficiency (1)	Adenovirus (1)	Pneumonia
Others (genetically not defined) (5)	HPV (2)	Warts
	EBV (4)	Hemophagocytosis (2), Viremia (1), Pneumonia (1)
	CMV (1)	Pneumonia
	Rhinovirus (1)	Pneumonia
	HSV (2)	Pneumonia (1), Stomatitis (1)
	Adenovirus (2)	Viremia (1), Enteritis (1)
**CONGENITAL DEFECTS OF PHAGOCYTE NUMBER OR FUNCTION (*****n*** **=** **1)**		
Glycogen storage disease type 1B	CMV (1)	Pneumonia
**COMPLEMENT DEFICIENCIES (*****n*** **=** **1)**		
Complement deficiency	H1N1 (1)	Pneumonia

*CMV, cytomegalovirus; EBV, Epstein–Barr virus; HHV-6, human herpesvirus 6; HSV, herpes simplex virus; HPV, human papilloma virus; VZV, varicella-zoster virus; RSV, respiratory syncytial virus*.

**Table 3:** Distribution of patients' diagnosis and viruses identified.

**Diagnosis organisms (*n*)**	**%**
**Viremia**	28.8
CMV (19)	
Adenovirus (12)	
EBV (10)	
Enteroviruses (6)	
HHV-6 (2)	
**Pneumonia**	28.2
CMV (18)	
Rhinovirus (9)	
Parainfluenza (5)	
Adenovirus (4)	
H1N1 (3)	
Coronavirus (2)	
RSV (2)	
EBV (2)	
Enterovirus (1)	
**Skin infections**	17.6
HPV (11)	
VZV (10)	
Molluscum contagiosum (9)	
**Gastrointestinal infections**	9.4
Norovirus (5)	
Enteroviruses (4)	
Adenovirus (2)	
CMV (2)	
Sapovirus (2)	
Rotavirus (1)	
**Stomatitis**	4.7
HSV (8)	
**Hemophagocytosis**	3.5
EBV (4)	
CMV (1)	
Enterovirus (1)	
**Meningitis**	2.4
EBV (1)	
CMV (1)	
HHV-6 (1)	
Enterovirus (1)	
**Retinitis**	2.4
CMV (4)	
**Keratitis**	1.2
HSV (2)	
**Myocarditis**	0.6
Poliovirus 2 (1)	
**Liver failure**	0.6
Adenovirus (1)	
**Lymphoproliferation**	0.6
EBV (1)	

*CMV, cytomegalovirus; EBV, Epstein–Barr virus; HHV-6, human herpesvirus 6; HSV, herpes simplex virus; HPV, human papilloma virus; VZV, varicella-zoster virus; RSV, respiratory syncytial virus*.

There were 80 deaths (29%) among the registered patients, 8 of them (10%) were caused by viral infections as follow: CMV pneumonia (2 patients), enterovirus sepsis (1 patient), RSV pneumonia (1 patient), poliovirus-2 myocarditis (1 patient), and adenovirus sepsis (3 patients, 1 of them complicated by liver failure).

## Discussion

In the current study, we present the characteristics of viral infections in a large cohort of PID children who were followed prospectively over a period of 15 years. Viral infections affected more than 1/3 of the registered patients, many of whom were affected by more than 1 virus. The patients were affected by a range of viral organisms but CMV, adenovirus and EBV were the culprits in almost half of the cases. The high frequency of CMV infections (>20%) in our cohort can be explained by the fact that most of the cases are affected by combined immunodeficiencies. Patients with such defects are extremely susceptible to progressive infection with CMV (12). Our finding that CMV and parainfluenza infections are more common in the group of immunodeficiencies affecting cellular and humoral immunity has been documented previously (7, 15). The observation that EBV is more common in the immune dysregulation group specifically triggering HLH is also well-documented (8, 12). We have found that patients with DOCK8 deficiency are particularly predisposed to mucocutaneous viral infections like molluscum contagiosum and HSV infections. This is probably since DOCK8 is an important regulator of the actin cytoskeleton that is critical for cell migration through collagen-dense tissue, hence playing an important antiviral immunity in the skin (16).

The presented cohort of patients are characterized by the high frequency of combined immunodeficiencies which are more severe with a higher predisposition to viral infections compared to other PID categories. Another prominent feature of our cohort is that none of the registered patients suffer from increased susceptibility to specific viral infections. Examples of such diseases are TLR3, TRIF, or UNC93B1 deficiencies which predispose to HSV-1 encephalitis and epidermodysplasia verruciformis or CXCR4 deficiencies which predispose to HPV.

It is important to stress that physicians should be aware of PIDs and consider them in patients with severe or recurrent viral infections. Importantly, they should be aware that many PIDs result in poor antibody response, hence serologic testing should be avoided while testing a patient for infectious complications and antigenic detection method should be used instead. Health care providers should also be aware of the recommendations for live viral vaccines in immunodeficient patients and their close contacts (17). Live vaccines, such as the chicken pox, measles, mumps, rubella (MMR), rotavirus, yellow fever, oral polio, and the influenza nasal spray should be avoided in certain types of PIDs. Furthermore, any infants born into a family with a suspicious history of PID should avoid all live viral and bacterial vaccines until PIDs is ruled out.

Historically, oral polio vaccine (OPV) was the only form used in the vaccination schedule in Kuwait. Since 2008 the first dose of OPV given at the age of 2 months was replaced with the inactivated formulation. Fortunately, only 1 patient from our cohort who was diagnosed with RAG1 deficiency developed OPV related complication (i.e., myocarditis). Two more patients with CID had prolonged excretion of poliovirus type 1 in the stool. Unfortunately, stool surveillance program of PID patients for vaccine derived polio virus is not available in the country.

The present study has some limitations since we did not determine the true burden of viral infections in PIDs. This could be established by documenting the number of admissions to the intensive care unit and the type of care provided like mechanical ventilation and the use of inotropes during these admissions. Other important variables that can be considered are the number of viral infection reactivations, the number of admissions to the hospital, the length of stay, and duration of using antiviral treatments. However, an important strength of the study is that the patients were followed prospectively by the same clinical immunologist. Another important strength of the study is that most patients were diagnosed at the molecular level. This may help in determining the genotype-phenotype correlation. Yet, collaborative efforts will be needed to collect a bigger number of patients.

Viral infections in PIDs should be treated aggressively with appropriate antiviral medications and definitive treatments like HSCT when possible since failure to eradicate viral pathogens creates an inflammatory environment that promotes cell survival and proliferation and may predispose to malignancy (18). Innovative treatments like virus-specific T cells should be explored to improve clinical outcomes for this group of patients (19).

## Data Availability

All datasets generated for this study are included in the manuscript and/or the supplementary files.

## Ethics Statement

The data was obtained from the Kuwait National Primary Immunodeficiency Disorders Registry (KNPIDR) which was approved by The Research and Ethics Committee of the Ministry of Health in Kuwait and the Kuwait University Health Sciences Center Ethical Committee in accordance with the Declaration of Helsinki.

## Author Contributions

WA-H: development of the research concept and goals, design of methodology, data collection and analysis, writing the initial manuscript draft, approval of the submitted manuscript, and agreement to be accountable for the content of the work. SE: contributed to the research idea, writing of the manuscript and approval of the submitted manuscript and agreement to be accountable for the content of the work.

### Conflict of Interest Statement

The authors declare that the research was conducted in the absence of any commercial or financial relationships that could be construed as a potential conflict of interest.
